# Impact of uterine adenomyosis on survival outcome of patients with non-endometrioid endometrial cancer

**DOI:** 10.1186/s12885-025-14815-4

**Published:** 2025-10-14

**Authors:** Levent Ozgen, Yakup Yalcin, Merve Abay, Kemal Ozerkan

**Affiliations:** Uludağ University, Bursa, Turkey

**Keywords:** Adenomyosis, Disease free survival, Non-endometrioid endometrial cancer, Overall survival

## Abstract

**Background:**

The impact of the presence of adenomyosis on survival in patients with non-endometrioid endometrial cancer (EC) remains unclear. The aim of this study is to compare the effect of the presence or absence of histologically proven adenomyosis on the survival of patients with non-endometrioid EC.

**Methods:**

We identified all patients who were consecutively diagnosed with non-endometrioid EC and underwent surgery in a single center between May 1998 and March 2023. Patients with insufficient clinical or surgical data were excluded from the study. A total of 139 non-endometrioid EC patients in the study were divided into two groups as with and without adenomyosis. Demographic characteristics and clinical findings such as age, BMI, menopausal status and pathologic variables such as presence of adenomyosis, tumor grade, depth of myometrial invasion, lymphovascular space involvement, lymph node status, and distant spread were obtained hospital records. Kaplan Meier analysis was performed for survival analysis. Overall (OS), and disease-free survival (DFS) were calculated.

**Results:**

A total of 139 patients, 40 (28.7%) in the adenomyosis group and 99 (71.3%) in the non-adenomyosis group, were included in the study and their data were recorded.There was no significant difference between patients with non-endometrioid type EC with and without adenomyosis in terms of patient demographic characteristics and pathological variables (*p* > 0.05).When the patients in the adenomyosis and non-adenomyosis groups were compared, there was no statistically significance regarding recurrence time (175.2 ± 24.4 months vs. 95.1 ± 11.2 months, *p* = 0.166). However, OS was found to be statistically significantly higher in patients with adenomyosis than in those without adenomyosis (172 ± 24.1 months vs. 102 ± 13.9 months; *p* = 0.02).

**Conclusions:**

The presence of adenomyosis in non-endometrioid type endometrial cancer was not associated with pathological variables such as myometrial invasion, tumor diameter and lympho-vascular space involvement. Although DFS and cancer-related death rates were similar, OS was significantly higher in the presence of adenomyosis.

**Supplementary Information:**

The online version contains supplementary material available at 10.1186/s12885-025-14815-4.

## Introduction

Endometrial cancer (EC) is the most common gynecological malignancy in developing countries. Its incidence in women is approximately 2.5-3%, and the mortality rate is reported to increase by an average of 1.9% per year. The standard approach to the treatment of endometrial cancer is surgery, including total hysterectomy with bilateral salpingo-oophorectomy and either sentinel node biopsies or lymphadenectomy in women at high risk for nodal metastases [1, 2].

Endometrial carcinomas include a variety of types with different prognosis and traditionally it has been classified according to its histomorphological features. Estrogen-induced type 1 endometrioid carcinomas (endometrioid type) are more common and account for 70–80% of cases, while non-estrogen-induced type 2 endometrioid carcinomas (non-endometrioid endometrial carcinomas) are less common and exhibit more aggressive behavior [3]. Serous, clear cell, carcinosarcoma, and undifferentiated carcinoma constitute the aggressive and poor-prognosis histologic subtypes of nonendometrioid endometrial carcinoma. This histologic heterogeneity leads to inconsistency in pathologist interpretation, delaying diagnosis and making management of this group more difficult. These subtypes are typically high-grade, exhibit a greater propensity for deep myometrial invasion, lymphovascular space invasion (LVSI), cervical stromal involvement, and extrauterine dissemination even at early stages. Consequently, patients with non-endometrioid EC often present with advanced-stage disease and have lower response rates to standard adjuvant therapies, resulting in reduced progression-free and overall survival compared to those with endometrioid tumors [4].

Adenomyosis is characterized by the presence of endometrial epithelium and stroma in the muscular layer of the uterus. The frequency of adenomyosis is around 20–30% in cases of hysterectomy performed for benign reasons and similarly 22.6% in patients with endometrial cancer [5]. The pathogenetic mechanisms in the development of endometrial cancer are similar to benign lesions such as endometriosis and adenomyosis. These common mechanisms include hormone dependency, genetic predisposition, similarity in growth and environmental factors, inflammation and alteration of the immune system [6].

The presence of adenomyosis, one of the most common additional histopathologic findings in endometrial cancer, has been found to be associated with better overall survival, earlier stage and lower grade, indicating a better prognosis in patients with endometrioid EC [7, 8]. However, there is still uncertainty about the relationship between the presence of adenomyosis and prognosis in patients with non-endometrioid EC [9, 10].

The aim of this study was to evaluate the effect of the presence of adenomyosis on overall survival (OS) and disease-free survival (DFS) in the less common but poorly progressive non-endometrioid EC.

## Materials and methods

This retrospective cross-sectional cohort study was carried out in the Department of Gynecologic Oncology at Bursa Uludag University Hospital. We identified all patients with consecutive diagnosis of EC who underwent surgery at a single center between May 1998 and March 2023.Within the scope of the study, the files of the patients and the information on the hospital information management system and the patients from the Ministry of Health Death Notification System were scanned retrospectively.

**Fig. 1:**
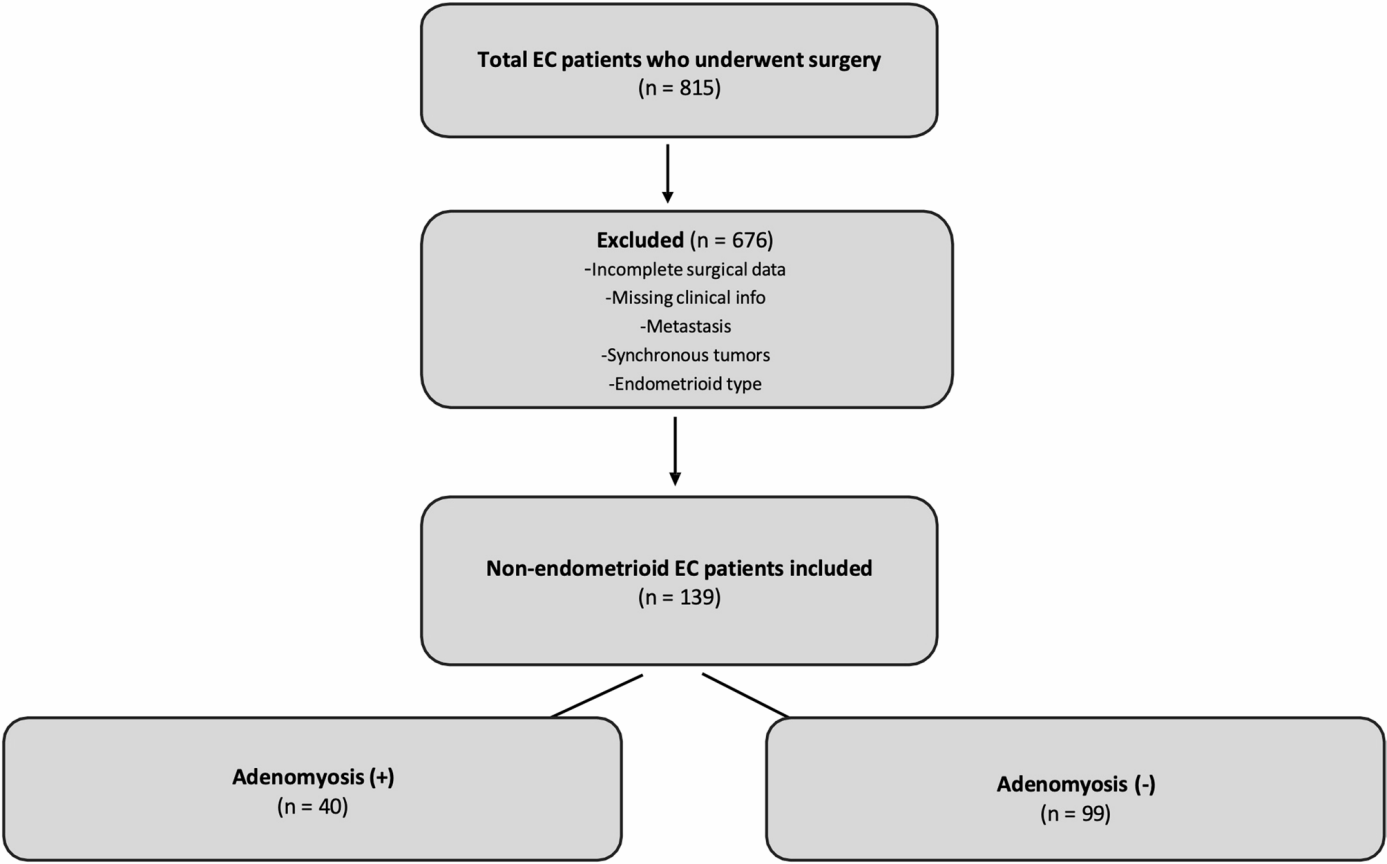
Flowchart of patient selection and study cohort

A total of 815 patients diagnosed with endometrial cancer (both endometrioid and non-endometrioid subtypes) were initially identified. Of these, 157 were excluded due to reasons such as inadequate surgical intervention, missing clinical or surgical data, metastasis to the endometrium from another primary tumor, presence of a synchronous malignancy, or a history of prior cancer surgery. Among the remaining 658 patients, 519 were excluded for having endometrioid-type cancer. Consequently, the study focused on 139 patients with non-endometrioid endometrial cancer who met the inclusion criteria and whose pathology reports were reviewed. The exclusion criteria and the study flow chart have been presented in Fig. 1 to provide a clear overview of the patient selection process and study design.

All pathological specimens were prepared and examined by the gynecological pathologists in our hospital. Patients were divided into 2 subgroups according to the presence or absence of adenomyotic tissue. Demographic characteristics of the patients, such as age, parity, body mass index (BMI), menopausal status, diabetes and hypertension, were documented from hospital records. The type of operation performed (hysterectomy, pelvic/paraaortic lymphadenectomy (+-) and omentectomy (+-), presence or absence of adjuvant therapy, chemotherapy (CT), radiotherapy (RT) and/or chemoradiotherapy (CRT) were recorded. Postoperative histopathological final diagnosis and the type of carcinoma, stage of the disease, tumor diameter, tumor grade, depth of myometrial invasion, cervical stromal invasion, lympho-vascular space invasion (LVSI), adnexal involvement, presence of lymph node involvement, peritoneal fluid cytology, preoperative cancer antigen CA-125 levels were evaluated and the information was recorded. Overall survival (OS), disease free survival (DFS), recurrence time and presence of mortality were evaluated using the hospital information system program and the data were recorded. Ethics committee approval and permission for the study was obtained by the local ethics committee of Bursa Uludağ University Faculty of Medicine Hospital (Date: 28/02/2022 Decision No: 2011-KAEK-26/118). Due to the retrospective design of this study, written informed consent forms were not obtained from the participants. The study was conducted in accordance with the principles of the Declaration of Helsinki.

### Statistical analysis

All statistical analyses were performed using IBM SPSS Statistics for Windows, Version 22.0 (IBM Corp., Armonk, NY, USA). Descriptive statistics were expressed as mean ± standard deviation and median (minimum-maximum) for continuous variables and as number (n) and percentage (%) for categorical variables. The distributional characteristics of continuous variables were assessed using the Shapiro-Wilk test. Group comparisons for continuous variables were made using the Independent Samples t-test when parametric assumptions were met and the Mann-Whitney U test when those assumptions were not met. Comparisons of categorical variables between groups were performed using the Chi-square test or Fisher’s exact test, as appropriate. Survival analyses were conducted using the Kaplan-Meier method, and survival curves were compared using the log-rank test. Univariate Cox proportional hazards regression analyses were performed to identify potential disease-free survival (DFS) and overall survival (OS) predictors. Variables with a *p*-value < 0.10 in the univariate analysis were included in the multivariate Cox regression model using the enter method to control for confounding effects. Hazard ratios (HRs) and 95% confidence intervals (CIs) were reported for each covariate. A Type I error rate of 5% was adopted.

## Results

Data of 139 patients who underwent surgery for non-endometrioid EC were analyzed in the study. It was determined that 28.7% (*n* = 40) of the patients had adenomyosis and 71.3% (*n* = 99) did not have adenomyosis.

Table 1 shows the demographic and clinical characteristics of the entire study population. The median age of the total population included in the study was 66 [40–88] years old, 97.8% (*n* = 136) were in the postmenopausal period. The median BMI of the total patient population was 32.6 [20-51.9] kg/m^2^. When the stage of the total patient group was examined, 38.8% were found to be stage 1a, 12.9% were stage 1b, 6.5% were stage 2 and 41.7% were stage 3 ≥ and above. The median value of preoperative Ca-125 levelin the totally study group was 27.4 (4-1885) IU/ml. Hysterectomy alone was performed in 12.2% of patients. 5.8% underwent hysterectomy and pelvic lymphadenectomy, and 82% underwent hysterectomy with pelvic/paraaortic lymphadenectomy. While 5.8% of the patients did not receive adjuvant treatment, 13.7% received CT, 24.5% received RT, and 56.1% received CRT. There was no significant difference between the groups with and without adenomyosis in terms of age, BMI, comorbidities, menopausal status, Ca-125 level, type of surgery performed, omentectomy, stage and adjuvant treatment (*p* > 0.05).

**Table 1:** Demographic characteristic findings and surgery type of patients

Characteristics	Total Population (*n* = 139) Median or number (%)	Adenomyosis (*n* = 40) Median or number (%)	Non-adenomyosis (*n* = 99) Median or number (%)	*P*
Age, years	66 (40–88)	65.5 (48–88)	66 (40–86)	0.885
BMI, kg/m^2^	32.6 (20-51.69)	33.5 (21–44)	32.5 (20-51.6)	0.235
Other diseases				
None	10 (7.19)	2 (5)	8 (8.1)	0.546
DM	11 (7.91)	4 (10)	7 (7.1)	
HT	73 (52.51)	24 (60)	49 (49.5)	
DM + HT	45 (32.37)	10 (25)	35 (35.4)	
Menapouse Status				
Postmenopausal	136 (97.8)	40 (100)	96 (97)	0.557
Premenopausal	3 (2.2)	0 (0)	3(3)	
CA-125, IU/L	24.7 (4-1885)	17.5 (7.2–330)	28.8 (4-1885)	0.119
Type of surgery				
Hysterectomy	17 (12.2)	4 (10)	13 (13.1)	0.756
Hysterectomy + PLND	8 (5.8)	3 (7.5)	5 (5.1)	
Hysterectomy + PPLND	114 (82)	33 (82.5)	81 (81.8)	
Omentectomy				
Yes	122 (87.8)	36 (90)	86(86.9)	0.778
No	17 (12.2)	4 (10)	13 (13.1)	
Stage				
1a	47 (38.8)	16 (40)	31 (31.3)	0.621
1b	25 (12,9)	5 (12.5)	20 (20.2)	
2	9 (6.5)	2 (5)	7 (7.1)	
*≥* 3	58 (41.7)	17 (42.5)	41 (41.4)	
Adjuvan treatment				
None	8 (5.8)	2 (5)	6 (6.1)	0.890
KT	19 (13.7)	8 (20)	11 (11.1)	
RT	34 (24.5)	9 (22.5)	25 (25.3)	
KRT	78 (56.1)	21 (52.5)	57 (57.6)	

Data in boldfont indicates statistically significant values

*BMI* Body Mass Index, *DM* diabetes mellitus, *HT* hypertension, *PLND* pelvic lymph node dissection, *PPLND* pelvic and para-aortic lymph node dissection, *KT* chemotherapy, *KRT* chemoradiotherapy

Data are given as median or n (%). *P* < 0.05 accepted as statistically significant

The most common histological type in the study group was serous EC with 38.8%. There was no difference between the groups in terms of histological types (*p* = 0.147). More than half of myometrial invasion was detected in 35% of the patients in the adenomyosis group and in 45.5% of the patients in the non-adenomyosis group. No statistically significant difference was found between both groups (*p* = 0.259). Similarly, no significant difference was found between adenomyotic and non-adenomyotic EC patients in the analysis of cervical stromal invasion, lympho-vascular space invasion, positive peritoneal fluid cytology, tumor size, omental metastasis and lymph node metastasis, which are poor prognostic indicators for EC (*p* = 0.926, *p* = 0.307, *p* = 0.511, *p* = 0.360, *p* = 0.238 and *p* = 0.717, respectively). The pathological findings of the patients are presented in Table 2.

**Table 2:** Pathological findings of patients

Characteristics	Total Population (*n* = 139) Median or number (%)	Adenomyosis (*n* = 40) Median or number (%)	Non-adenomyosis (*n* = 99) Median or number (%)	*P*
Hystological type				
Serous	54 (38.8)	16 (40)	38 (38.4)	0.147
Mucinous	14 (10.1)	5 (12.5)	9 (9.1)	
Clear cell	17 (12.2)	5 (12.5)	12 (12.1)	
Carcinocarcoma	31 (22.3)	4 (10)	27 (27.3)	
Undifferentiated	16 (11.5)	8 (20)	8 (8.1)	
Mixt type	7 (5)	2 (5)	5 (5.1)	
Myometrial invasion				
< 1/2	80 (57.6)	26 (65)	54 (54.5)	0.259
*≥* 1/2	59 (42.4)	14 (35)	45 (45.5)	
Cervical stromal invasion				
Yes	32 (23)	9 (22.5)	23 (23.2)	0.926
No	107 (77)	31 (77.5)	76 (76.8)	
Lymphovascular space invasion				
Yes	81 (58.3)	26 (65)	55 (55.6)	0.307
No	58 (41.7)	14 (35)	44 (44.4)	
Tumor size, cm	4 (0.4–15.3)	3.75 (0.5–10.5)	4 (0.4–15.3)	0.360
Peritoneal fluid cytology				
Negative	128 (92.1)	38 (95)	90 (90.9)	0.511
Positive	11 (7.9)	2 (5)	9 (9.1)	
Omental metastasis				
Yes	16 (11.5)	7 (17.5)	9 (9.1)	0.238
No	123 (88.5)	33 (82.5)	90 (90.9)	
Lymph node metastasis				
No	104 (74.8)	33 (82.5)	71 (71.7)	0.717
Pelvic	6 (4.3)	1 (2.5)	5 (5.1)	
Paraaortic	5 (3.6)	1 (2.5)	4 (4)	
Pelvic + paraaortic	24 (17.3)	5 (12.5)	19 (19.2)	

Data are given as median or n (%). *P* < 0.05 accepted as statistically significant

Univariate Cox regression analyses were performed to identify factors associated with disease recurrence. The results showed that stage (*p* = 0.015), tumor size (*p* = 0.109), presence of lymphovascular space invasion (LVSI) (*p* = 0.081), presence of cervical stromal invasion (*p* = 0.049), lymphatic metastasis (*p* = 0.002), and presence of omental metastasis (*p* = 0.086) were statistically associated with recurrence. However, in the multivariate analysis, none of the variables that were found to be significant in the univariate analysis retained a statistically significant association with recurrence (*p* > 0.05) (Table 3).

**Table 3:** Univariate and multivariate analysis of factors affecting disease-free survival

	Univariate Model		Mulivariable Model	
	HR(95%CI)	*p*	HR(95%CI)	*p*
Age (years)	1.010 (0.984–1.037)	0.458	–	–
BMI (kg/m2)	1.016 (0.977–1.056)	0.427	–	–
Group (Adenomyosis)	0.680 (0.392–1.181)	0.171	–	–
Menopause (Yes)	2.698 (0.374–19.485)	0.325	–	–
Comorbididity		0.315	–	–
• DM (Ref: No)	0.425 (0.095–1.905)	0.264	–	–
• HT (Ref: No)	1.276 (0.457–3.563)	0.642	–	–
• DM & HT (Ref: No)	1.094 (0.371–3.228)	0.870	–	–
CA125	1.000 (0.999–1.001)	0.743	–	–
Histologic Type		0.278	–	–
• Musinous - Serous (Ref.)	0.604 (0.245–1.489)	0.274	–	–
• Clear Cell - Serous (Ref.)	1.182 (0.562–2.490)	0.659	–	–
• Carcinosarcoma - Serous (Ref.)	1.597 (0.866–2.943)	0.134	–	–
• Undifferentiated - Serous (Ref.)	0.751 (0.321–1.755)	0.508	–	–
• Mixed - Serous (Ref.)	0.731 (0.219–2.443)	0.611	–	–
Stage		0.015		0.454
• IB – IA (Ref.)	1.023 (0.458–2.283)	0.957	0.999 (0.444–2.248)	0.998
• II – IA (Ref.)	2.144 (0.829–5.547)	0.116	2.348 (0.786–7.017)	0.126
• III – IA (Ref.)	2.306 (1.264–4.206)	0.006	0.976 (0.384–2.482)	0.959
Tumor Size (cm)	1.085 (0.982–1.200)	0.109	1.027 (0.902–1.169)	0.690
Myometrial Invasion (> 50%)	1.226 (0.761–1.976)	0.403	–	–
LVSI (Present)	1.533 (0.949–2.475)	0.081	1.197 (0.660–2.173)	0.554
Cervical Stromal Invasion (Present)	1.692 (1.001–2.860)	0.049	0.962 (0.495–1.871)	0.909
Peritoneal Cytology (Positive)	0.955 (0.384–2.374)	0.921	–	–
Surgical Approach – Laparotomy	0.526 (0.210–1.317)	0.170	–	–
Surgery		0.600	–	–
• Hysterectomy & PLND – Hysterectomy (Ref.)	1.137 (0.360–3.588)	0.826	–	–
• Hysterectomy & PPLND – Hysterectomy (Ref.)	0.775 (0.405–1.485)	0.443	–	–
Lymph Node Metastasis		0.002		0.092
• Pelvic – (Ref: No)	2.570 (0.919–7.184)	0.072	3.028 (0.859–10.678)	0.085
• Paraaortic – (Ref: No)	1.805 (0.559–5.829)	0.323	2.388 (0.573–9.961)	0.232
• Pelvic&Paraaortic – (Ref: No)	2.882 (1.632–5.090)	< 0.001	2.956 (1.237–7.061)	0.015
Adjuvant Treatment		0.244	–	–
• KT – (Ref: No)	0.848 (0.313–2.299)	0.746	–	–
• RT – (Ref: No)	0.462 (0.176–1.209)	0.116	–	–
Omentectomy (Performed)	0.793 (0.416–1.512)	0.481	–	–
Omental Metastasis (Present)	1.761 (0.923–3.361)	0.086	2.038 (0.872–4.761)	0.100

*HR* Hazard Ratio, *CI* Confidence Interval, *Ref* Reference Category

Univariate Cox regression analyses were conducted to identify factors affecting overall survival. The results revealed that the presence of adenomyosis (*p* = 0.024), cancer subtype (*p* = 0.041), stage (*p* = 0.002), tumor size (*p* = 0.010), presence of lymphovascular space invasion (LVSI) (*p* = 0.006), presence of cervical stromal invasion (*p* = 0.008), lymphatic metastasis (*p* = 0.001), presence of omental metastasis (*p* = 0.016), and the development of recurrence during follow-up (*p* < 0.001) were significantly associated with overall survival. However, in the multivariate analysis, only the development of recurrence during follow-up remained an independent predictor of overall survival. The risk of death in patients who developed recurrence was 8.36 times higher than in those without recurrence (HR = 8.360; 95% CI: 4.412–15.841; *p* < 0.001). Despite being included in the model, the other variables did not have a statistically significant impact on overall survival (*p* > 0.05) (Table 4).

**Table 4:** Univariate and multivariate analysis of factors affecting overall survival

	Univariate Model		Mulivariable Model	
	HR(95%CI)	*p*	HR(95%CI)	*p*
Age (years)	1.018 (0.993–1.043)	0.158	-	-
BMI (kg/m2)	1.011 (0.974–1.050)	0.550	-	-
Group (Adenomyosis)	1.864 (1.086–3.199)	0.024	0.877 (0.477–1.610)	0.671
Menopause (Yes)	1.675 (0.410–6.841)	0.473	-	-
Comorbididity	–	0.520	-	-
• DM (Ref: No)	1.112 (0.417–2.960)	0.832	-	-
• HT (Ref: No)	0.606 (0.228–1.610)	0.315	-	-
• DM & HT (Ref: No)	1.194 (0.713–1.998)	0.500	-	-
CA125	1.000 (0.999–1.001)	0.910	-	-
Histologic Type	–	0.041	-	-
• Musinous - Serous (Ref.)	0.404 (0.164–0.992)	0.048	0.633 (0.230–1.742)	0.376
• Clear Cell - Serous (Ref.)	0.995 (0.495–2.002)	0.989	1.044 (0.482–2.262)	0.914
• Carcinosarcoma - Serous (Ref.)	1.624 (0.924–2.855)	0.092	1.558 (0.825–2.944)	0.172
• Undifferentiated - Serous (Ref.)	0.704 (0.330–1.498)	0.362	0.659 (0.282–1.542)	0.336
• Mixed - Serous (Ref.)	0.498 (0.149–1.661)	0.257	0.481 (0.130–1.775)	0.272
Stage	–	0.002	-	-
• IB – IA (Ref.)	0.946 (0.441–2.028)	0.886	0.870 (0.395–1.915)	0.729
• II – IA (Ref.)	2.035 (0.799–5.184)	0.136	1.351 (0.471–3.880)	0.576
• III– IA (Ref.)	2.466 (1.409–4.317)	0.002	1.069 (0.453–2.521)	0.879
Tumor Size (cm)	1.133 (1.031–1.246)	0.010	1.006 (0.875–1.156)	0.934
Myometrial Invasion (> 50%)	1.216 (0.780–1.894)	0.388	-	-
LVSI (Present)	1.864 (1.196–2.905)	0.006	1.728 (0.964–3.099)	0.066
Cervical Stromal Invasion (Present)	1.920 (1.182–3.118)	0.008	1.274 (0.670–2.423)	0.461
Peritoneal Cytology (Positive)	1.413 (0.679–2.939)	0.355	-	-
Surgical Approach – Laparotomy	0.987 (0.310–3.146)	0.982	-	-
Surgery	–	0.490	-	-
• Hysterectomy & PLND – Hysterectomy (Ref.)	1.150 (0.373–3.547)	0.808	-	-
• Hysterectomy & PPLND – Hysterectomy (Ref.)	0.749 (0.412–1.364)	0.345	-	-
Lymph Node Metastasis	–	0.001	-	-
• Pelvic – (Ref: No)	2.164 (0.779–6.015)	0.139	3.505 (0.966–12.716)	0.056
• Paraaortic – (Ref: No)	0.909 (0.221–3.736)	0.895	0.609 (0.123–3.012)	0.543
• Pelvic&Paraaortic – (Ref: No)	2.885 (1.685–4.941)	< 0.001	1.313 (0.589–2.924)	0.506
Adjuvant Treatment	–	0.173	-	-
• KT – (Ref: No)	1.237 (0.440–3.482)	0.687	-	-
• RT – (Ref: No)	0.647 (0.239–1.750)	0.391	-	-
Omentectomy (Performed)	1.306 (0.720–2.371)	0.380	-	-
Omental Metastasis (Present)	2.041 (1.142–3.649)	0.016	1.971 (0.924–4.205)	0.079
Recurrence (Yes)	8.192 (4.581–14.650)	< 0.001	8.360 (4.412–15.841)	< 0.001

*HR* Hazard Ratio, *CI* Confidence Interval, *Ref* Reference Category

**Fig. 2:**
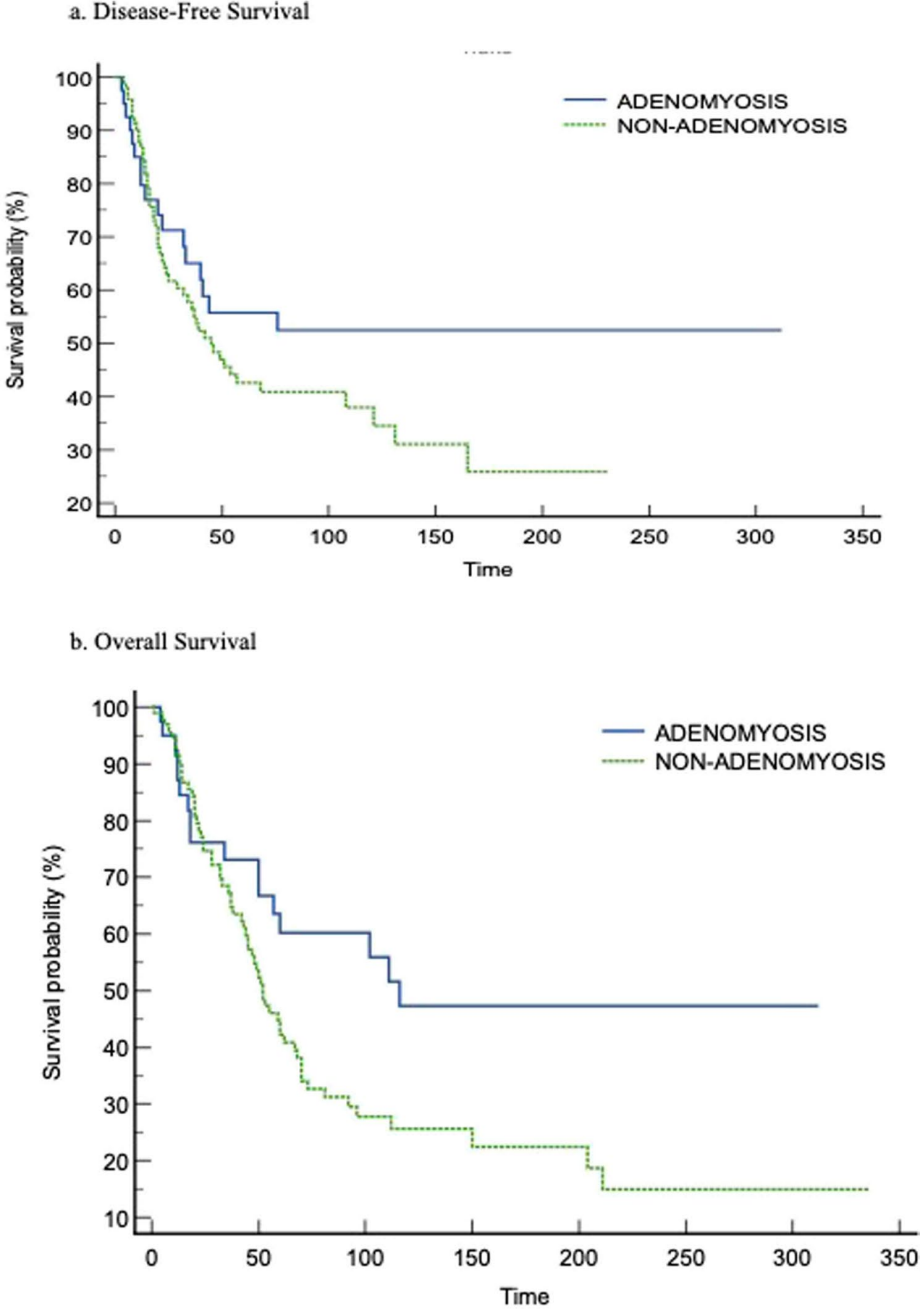
Disease-free survival (2a) and overall survival (2b) results in patients with endometrial cancer in the adenomyosis and non-adenomyosis groups

Mortality occurred in 56.83% (*n* = 79) of the total population during follow-up in the study group. While 78.4% (*n* = 62) of these patients were in the non-adenomyosis group, 21.6% (*n* = 17) were in the adenomyosis group. The difference was not statistically significant (*p* = 0.537). The mean overall survival time of the total study group during follow-up was 125.1 ± 13.1 months while this period was 172 ± 24.1 months in the adenomyosis group and 102 ± 13.9 months in the non-adenomyosis group. There was a statistically significant difference between the groups in favor of EC patients with adenomyosis coexistence (*p* = 0.021). The duration of recurrence in the total group was 137 ± 13.8 months. Although this period was longer than the mean recurrence time of 175.2 ± 24.4 months in the group with adenomyosis and 95.1 ± 11.2 months in the non-adenomyotic group, it did not make a statistically significant difference (*p* = 0.166). Disease-free survival by time in adenomyotic and non-adenomyotic groups in patients with EC is shown in Fig. 2a, and overall survival is shown in Fig. 2b.

## Discussion

In our study, the presence of adenomyosis was not associated with a statistically significant difference in disease-free survival (DFS); however, it was significantly associated with improved overall survival (OS). This finding suggests that adenomyosis may not directly reduce the risk of tumor recurrence but rather may be associated with certain biological or clinical features that make the disease more manageable, thus contributing to long-term survival. Several hypotheses have been proposed to explain the good prognosis in EC patients with adenomyosis. Increased levels of the antitumoral cytokines such as interferon-γ, tumor necrosis factor-α and interleukin 10 in adenomyosis tissue and decreased levels of oncogenic cytokines interleukin 1-beta, interleukin 8, epidermal growth factor and transforming growth factor create a protective effect. Another hypothesis is that adenomyosis blocks cancer invasion by mechanically contributing to the surrounding hypertrophic and hyperplastic myometrial stroma [9]. On the contrary, some mechanisms may support the poor prognostic effect of adenomyosis. One of them is that the large contact area between the ectopic endometrium layer and the uterine muscle layer in adenomyosis can increase the depth of myometrial infiltration of EC. The other is that ectopic endometrium can increase the invasion of the lympho-vascular area by following the mechanism of spread through lymph and vessels [11]. The literature also provides evidence for direct malignant transformation of adenomyosis. Endometrial Cancer caused by adenomyosis, which constitutes less than 1% of EC, originates from the epithelium of adenomyotic foci located between the muscle fibers of the uterine myometrium. A comparison of endometrial cancer arising in adenomyosis (EC-AIA) and endometrial cancer coexisting with adenomyosis (EC-A) concluded that EC-AIA is associated with poor survival [9, 11–13]. Our study did not include patients with endometrial cancer arising in adenomyosis (EC-AIA).

The clinical significance of the association of adenomyosis and endometrial cancer has not been clearly established. Although the presence of adenomyosis has been shown to be associated with better overall survival, earlier stage and lower FIGO grade in patients with endometrioid type EC, there is still uncertainty about the prognosis for patients with non-endometrioid endometrial cancer. To our knowledge, there are a few studies in the literature on the relationship between non-endometrioid EC and adenomyosis [14–16]. Due to the limitations of studies in the literature, in this study we tried to determine whether the presence of adenomyosis has a positive or negative prognostic effect on OS and DFS in patients with non-endometrioid EC.

According to Raffone et al.’s analysis of eight retrospective cohort studies evaluating 5573 EC patients to better understand the prevalence, histological association and relationship between endometrial carcinoma and adenomyosis, the pooled adenomyosis prevalence was found to be 22.6%. This result revealed that the prevalence of adenomyosis in EC patients was not different from that reported for other gynecological conditions. Also, EC patients with adenomyosis have a significantly reduced risk for adverse histologic prognostic factors of EC compared to EC patients without adenomyosis. Such findings may explain the proposed better EC prognosis in patients with adenomyosis [5].

In a recent meta-analysis by An et al., based on 14 retrospective observational studies including 1308 patients withEC with adenomyosis and 3734 patients with ECwithout adenomyosis, a positive increase in OS was observed in patients with adenomyosis, but no difference in DFS was observed between the two groups. It was also observed that patients with adenomyosis were younger, had less deep MI, less LVSI positivity, and more grade 1 and FIGO I-II stages. It has been reported that there is no significant difference in terms of histologic type and lymph node metastasis [17]. In our study, no difference was found in terms of prognostic factors.

The results of another meta-analysis showed that it significantly increased OS in EC patients with concomitant adenomyosis, but unlike most studies, there was no difference in DFS. However, some of the studies comprising this meta-analysis included only EC patients with endometrioid histologic type. Since endometrioid histologic type is the group with the best prognosis in EC patients, the results of the current meta-analysis may have been affected by this patient selection [11].

Matsuo et al., in their study including stage 1–4 EC patients, stated that the adenomyosis group was at an earlier stage and the probability of myometrial invasion and cervical invasion was lower. Furthermore, significantly better DFS and OS were reported in the EC group with adenomyosis in this study. When both disease-free survival and overall survival time between the endometrioid and non-endometrioid groups were evaluated, it was found that both were better in the endometrioid group. An independent effect of adenomyosis could not be demonstrated in multivariate analysis. No subgroup analysis was performed in terms of survival between patients with and without adenomyosis in the non-endometrioid group [14]. In our study, the OS in the adenomyosis group was 172 ± 24.1 months, which was significantly better than the mean OS of the non-adenomyotic EC group, which was 102 ± 13.9 months. However, we did not detect any difference in DFS between the adenomyotic and non-adenomyotic groups in our study.

Sanci et al. evaluated the effect of adenomyosis on overall survival and prognostic histopathological factors in 400 EC patients. The study found no difference between the 2 groups in terms of disease-free survival and death from cancer. However, multivariate analysis revealed an independent positive effect of adenomyosis on overall survival [16]. Since the number of non-endometrioid patients in the study was only 25, adenomyosis and non-adenomyosis subgroup analysis could not be performed in this group.

In the study of Çelik et al., both endometrioid and non-endometrioid EC types were included. Sub-analysis was performed to show the impact of different histological types on the results and a significant difference in the reduction of mortality was found in endometrioid type EC. There was no difference in disease-free survival and disease-related death between non-endometrioid type EC with and without adenomyosis. Disease-specific survival was significantly longer in patients with adenomyosis in the entire patient group, but progression-free survival was not affected by the presence of adenomyosis. These patients with coexistent adenomyosis and EC had better clinicopathological features and less advanced tumors. However, in multivariate analysis, adenomyosis was not found to be an independent prognostic factor for EC [18]. In our study, there was no significant difference between the adenomyosis group and the non-adenomyosis group in terms of these negative prognostic indicators.

One of the strengths of our study is that it includes a larger number of patients compared to studies in the literature where the number of patients with non-endometrioid endometrial cancer is limited. A significant contribution has been made to the literature on this subject, which contains contradictory evidence. All patients were operated on at a single center and pathological samples were evaluated by experienced gynecological pathologists. The retrospective design can be considered a limitation of our study.

## Conclusion

In conclusion, this study showed that the presence of adenomyosis was only significantly associated with higher overall survival in non-endometrioid EC. This result revealed in our study highlights that the presence of adenomyosis should be considered as a good prognostic factor in all EC types, including non-endometrioid endometrial cancer.

## Supplementary Information

Supplementary Material 1.

## Data Availability

“Data is provided within the supplementary information files”.
